# Conjugation of nitrated acetaminophen to Der p1 amplifies peripheral blood monocyte response to Der p1

**DOI:** 10.1371/journal.pone.0188614

**Published:** 2017-12-11

**Authors:** Ryan G. Thomas, Brenda M. Rivera Reyes, Benjamin M. Gaston, Nelki B. Rivera Acosta, Ilya R. Bederman, Laura A. Smith, Morgan T. Sutton, Benlian Wang, John F. Hunt, Tracey L. Bonfield

**Affiliations:** 1 Department of Pediatrics, Division of Pulmonology, University Hospitals Cleveland Medical Center, Rainbow Babies and Children’s Hospital, Cleveland, Ohio, United States of America; 2 Department of Pediatrics, Division of Pulmonology, Case Western Reserve University, Cleveland, United States of America; 3 Center of Proteomics and Bioinformatics, Case Western Reserve University, Cleveland, Ohio, United States of America; 4 Airbase Therapeutics, Charlottesville, Virginia, United States of America; Boston University, UNITED STATES

## Abstract

**Background:**

An association of acetaminophen use and asthma was observed in the International Study of Asthma and Allergies in Childhood study. However there are no clear mechanisms to explain an association between acetaminophen use and immunologic pathology. In acidic conditions like those in the stomach and inflamed airway, tyrosine residues are nitrated by nitrous and peroxynitrous acids. The resulting nitrotyrosine is structurally similar to 2,4-dinitrophenol and 2,4-dinitrochlorobenzene, known haptens that enhance immune responses by covalently binding proteins. Nitrated acetaminophen shares similar molecular structure.

**Objective:**

We hypothesized the acetaminophen phenol ring undergoes nitration under acidic conditions, producing 3-nitro-acetaminophen which augments allergic responses by acting as a hapten for environmental allergens.

**Methods:**

3-nitro-acetaminophen was formed from acetaminophen in the presence of acidified nitrite, purified by high performance liquid chromatography, and assayed by gas-chromatography mass spectrometry. Purified 3-nitro-acetaminophen was reacted with Dermatophagoides pteronyssinus (Der p1) and analyzed by mass spectrometry to identify the modification site. Human peripheral blood mononuclear cells proliferation response was measured in response to 3-nitro-acetaminophen and to 3-nitro-acetaminophen-modified Der p1.

**Results:**

Acetaminophen was modified by nitrous acid forming 3-nitro-acetaminophen over a range of different acidic conditions consistent with airway inflammation and stomach acidity. The Der p1 protein-hapten adduct creation was confirmed by liquid chromatography-mass spectrometry proteomics modifying cysteine 132. Peripheral blood mononuclear cells exposed to 3-nitro-acetaminophen-modified Der p1 had increased proliferation and cytokine production compared to acetaminophen and Der p1 alone (n = 7; p < 0.05).

**Conclusion:**

These data suggests 3-nitro-acetaminophen formation and reaction with Der p1 provides a mechanism by which stomach acid or infection-induced low airway pH in patients could enhance the allergic response to proteins such as Der p1.

## Introduction

Multivariate analysis of the International Study of Asthma and Allergies in Childhood (ISAAC) shows a dose dependent association between parental reported use of acetaminophen (AP) for fever in the first year of life and an increased risk of asthma, rhinoconjunctivitis, and eczema at 6–7 [1]. Similar results were found in the follow-up ISAAC analysis of 13–14 year old patients [2], a birth cohort in Ethiopia [3], and in adults [4].

Two important objections have been raised to AP being causally associated with asthma. First, the infections causing fever may also be casually associated with asthma [5]. Sordillo and coworkers showed the association of AP use for fever and asthma was not significantly greater than ibuprofen when controlling for infections [6]. An Asthmanet study [7] also failed to find an increased risk of asthma in children randomized to AP versus ibuprofen for fever. These studies emphasize infection as an important contributor to asthma risk.

The second objection has been the absence of a mechanism for this effect. One theory is AP depletes intracellular glutathione stores. Studies have shown up to a 53% decrease in glutathione stores in the lungs following a dose of AP [8]. However lower respiratory tract glutathione levels are 140 fold higher than serum [9] and glutathione resynthesis time is short [10]. Furthermore, airway glutathione in asthmatics is elevated [11,12] compared to controls. Importantly, the animal studies showing pulmonary depletion of gluathione after AP administration used toxic doses [13]. Glutathione depletion by AP seems unlikely to impact antioxidant defenses in the lung sufficiently to induce asthma/atopy.

**Fig 1:**
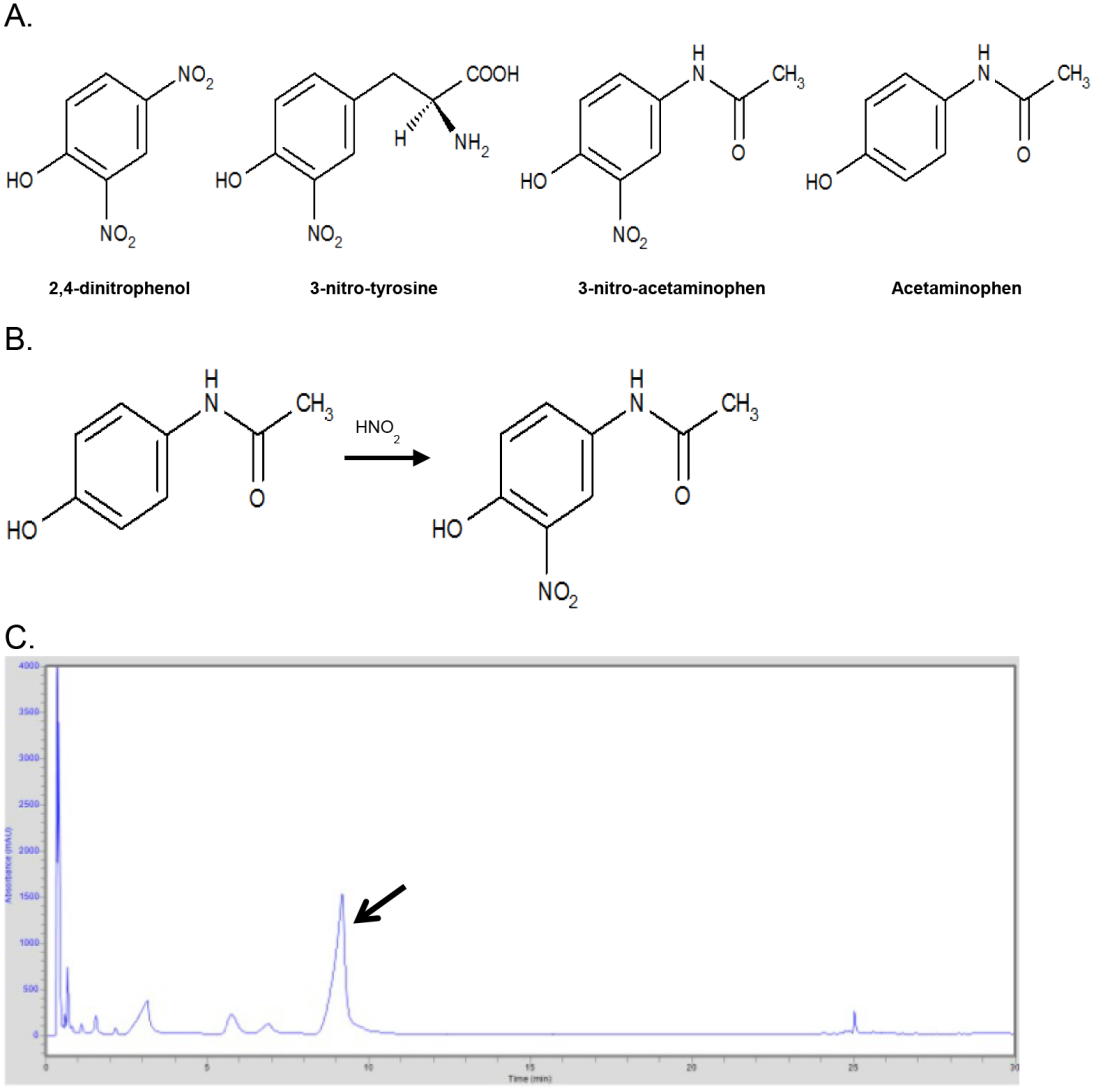
A. Structural simularity of 3-nitro-tyrosine and 3-nitro-acetaminophen (NAP) with 2,4-dinitrophenol, a well-known hapten. Acetaminophen shown for reference. B. Depiction of acetaminophen nitration reaction. C. High Performance Liquid Chromatography (HPLC) elution profile of NAP. Synthesized NAP was purified by HPLC, arrow denotes the NAP peak that is seen at elution time of 8 minutes. Figure is representative of results of multiple HPLC elutions and representative of similar profiles obtained at different pH.

We hypothesized that the combination of AP and inflammation amplifies the immune response to antigens. The inflamed airway is acidic [14–19]. When protonated, nitrite and peroxynitrite form the potent nitrating agents nitrous acid and peroxynitrous acid which readily nitrate tyrosine residues in endogenous and exogenous proteins. 3-nitro-tyrosine has been identified in inflamed tissue in asthma [20], cystic fibrosis [21, 22], allergic rhinitis [23] and chronic obstructive pulmonary disease [24] and is an in vivo marker of nitrative stress [25]. The chemical structure of 3-nitro-tyrosine is similar to those of 2,4-dinitrophenol and 2,4-dinitrochlorobenzene, both highly potent haptens [26–28] known for enhancing immune responses by binding to proteins (Fig 1). Immune response to environmental allergens can be amplified by tyrosine nitration. Nitration of the birch tree pollen allergen Bet v 1 enhanced the presentation of Bet v 1-derived peptides by human dendritic cells [29].

Like tyrosine and the pre-haptens discussed above, AP is a phenol readily nitrated under mildly acidic conditions to form stable 3-nitro-acetaminophen (NAP) [30]. Indeed, this reaction is the basis of the assay kit for serum AP levels [31]. NAP’s chemical structure is similar to the above haptens (Fig 1). Here, we show NAP is formed in vitro under conditions similar to those of the stomach or acidic airway in vivo and modifies Der p1, amplifying in vitro immune responses to it.

## Materials and methods

### Reagents

Unless noted, all reagents were purchased from Sigma Aldrich (St. Louis, MO).

### Synthesis of NAP

We synthesized NAP from AP (Sigma, St. Louis, MO) by mixing 6.25mL of 26.5nM AP with 5 mL of 6N HCL and 5 mL of 1.4M NaNO_2_ and 5 mL of 1.3M ammonium sulfamate [32]. HCl was used to acidify, NaNO2 was used as the nitrating agent, and the ammonium sulfamate was used to neutralize the excess nitrous acid. *Nitroparacetamol Reaction*: 200mg of acetaminophen was dissolved in 2 mL of methanol, and evaporated with the final residue dissolved in 50 mL H_2_0. To the dissolved AP, we added 0.25 mL of 1N, 0.1N, 0.05N, 0.01N, or 0.005N HCl which was followed by the addition of 0.25 mL of 1.4 mol/L NaN02. Each reaction mixture was neutralized with 0.25 mL 1.3 mol/L ammonium sulfamate. The reaction product was compared to nitroparacetamol stock standard.

### High performance liquid chromatography (HPLC) protocol for NAP Isolation

NAP was purified using the Flexar PSA PLUSS HPLC system, (PerkinElmer, Waltham, MA) using a Pecosphere C18 3mcM, 33x4.8mm column (PerkinElmer, Waltham, MA) and Chromera 4.1.0 software (PerkinElmer, Waltham, MA). The mobile phase with 20mM ammonium acetate buffer and acetonitrile. The detector was set at 245nm wavelength.

### Derivation and analysis

NAP identification by derivatization and gas chromotography-mass spectrometry (GC/MS; Agilent 6890; Santa Clara, CA). Lyophilized HPLC elutes were reacted with 80 μl of bis(trimethylsilyl) trifluoroacetamide + 10% trimethylchlorosilane (Regis, Morton Grove, IL) for 30 min at 75°C. NAP was identified using an Agilent 5973N-MSD equipped with an Agilent 6890 GC system (GC/MS). A DB17-MS capillary column (30 m × 0.25 mm × 0.25 μm) was used in all assays with a helium flow of 1 ml/min. Samples were analyzed in Scan mode using electron impact ionization (EI). Ion dwell time was set to 10 msec [33]. Samples were analyzed with electron impact ionization. The National Institute of Standards and Technology (NIST, Gaithersburg, MD) Mass spectral library with search program was used with AP as the standard for verification. The sensitivity of the nitration process to various acidic conditions was verified using Thermo Fisher TSQ triple-quad LC/MS instrument with direct injection in scan mode.

### Conjugation of NAP with Derp1 and purification and liquid chromatography mass spectrophotometry proteomics

Purified NAP (10mcg/mL) was reacted with Derp1 (Indoor Biotechnologies, Charlottesville, VA) (5mcg/mL) at in a 2:1 ratio at pH values of 7, 6 and 4. Samples were incubated at 37°C for 1 hour. The post-reaction Der p1 and NAP mixture was visualized on SDS-PAGE gel with Coomassie staining. SDS PAGE was washed in distilled water to remove excess background stain; the gel band corresponding to Der p1 was excised and cut into 1 mm^2^ pieces. The gel pieces were de-stained with 500 mcL of 1:1 acetonitrile and 100 mM ammonium bicarbonate solution for 2–8 hours. Afterwards, 10 mM reductive tris (2-carboxyethyl) phosphine was added and free cysteines alkylated with 55 mM iodoacetamide. Acetonitrile and 100 mM ammonium bicarbonate were used to dehydrate and rehydrate the gel pieces alternatively for three times. Gel pieces were swelled in 50 mM ammonium bicarbonate containing 10 ng/μL trypsin (Promega, Madison, WI), and Glu-C and digested overnight. Peptides were extracted with 60% acetonitrile/5% formic acid and dried in SpeedVac.

### Protein digestion

Gel pieces of PEPT1 protein bands cut from SDS-PAGE were destained with 50% acetonitrile in 100 mM ammonium bicarbonate, and then in 100% acetonitrile. The proteins in the gels were reduced by 20 mM 1,4-Dithiothreitol at room temperature for 30 min followed by alkylation by 50 mM idoacetamide in 100 mM ammonium bicarbonate for 30 min in the dark. After the treatment, the reagents were removed and the gel pieces were washed with 100 mM ammonium bicarbonate and then dehydrated in acetonitrile. The dried gel pieces were then reswelled in sequencing grade modified trypsin in 50 mM ammonium bicarbonate for overnight digestion. Tryptic peptides were extracted from the gel with 50% acetonitrile in 5% formic acid.

### Liquid chromatography -Tandem mass spectrometry (LC-MS/MS) proteomic analysis

Proteolytic peptide analysis was performed using Orbitrap Elite Hybrid Mass Spectrometer (Thermo Electron, San Jose, CA) equipped with a Waters nanoAcquity UPLC system (Waters, Taunton, MA). A full scan at 120,000 resolution was obtained in the Oribtrap for eluted peptides in the range of 300–1800 Da followed by MS/MS scans on the twenty most abundant precursor ions by collision-induced dissociation at normalized collision energy of 35%. Raw LC-MS/MS data were searched through Mascot search engine (version 2.2.0, Matrix Science) against PEPT1 protein primary sequence with Met oxidation, Cys carbamidomethylation and NAP modification as variable modifications. The mass tolerance was set as 10 ppm for precursor ion and 0.8 Da for product ion. The significance threshold was P < 0.05.

### Peripheral blood mononuclear cell (PBMC) stimulation

Whole blood was collected from healthy adult volunteers not currently taking AP, ibuprofen, or any antihistamine and placed in ethylenediaminetetraacetic acid (EDTA). PBMC’s were isolated by density gradient centrifugation using Ficoll-Paque PLUS (GE Healthcare Uppsala, Sweden), per the manufacturer’s instructions. The cells were washed, counted and plated at a density of 1x10^6^ cells/well in 1mL RPMI plus 10% heat inactivated fetal bovine serum. Treatment groups included no treatment (negative control), phytohemagluttin, to induce non-specific activation of T cells [34], (PHA, 50 ng/mL, positive control), Der p1 (50 ng/mL), AP (50ng/ml), NAP (50 ng/mL), Der p1 with NAP (50 ng/mL each), Der p1 mixed with AP (50 ng/mL each), Der p1 conjugated with NAP (50 ng/mL), or Der p1 conjugated with AP (50 ng/mL). These treatments were incubated at 37°C for 18 hours in 5% CO_2_, pH 7.4. Supernatant and cells were harvested after culture. ATP was measured in mol/mL with BacTiterGlo chemiluminescence assay (Promega, Madison, WI) as a surrogate for cellular activity [35] and therefore growth kinetics [36]. To account for intrasubject variability the data is presented as a ratio with negative control as the denominator. Cytokine (TNFa, IL-1β, IL-4, IL-5, IL-6, IL-10, IL-13, and IL-17) levels were measured in pg/mL with Multiplex Luminex assays (ThermoFischer, Waltham, MA).

### Statistics

Data are expressed as mean ± standard deviation. Comparisons of mean data were made through analysis of variance, and subsequent multiple comparisons were made using Bonferoni correction. A value of p< 0.05 was considered significant.

### Approvals

All studies involving human use were approved by the Institutional Review Board of University Hospitals Cleveland Medical Center (IRB#: 01-16-08) Informed consent was obtained for the blood draws.

## Results

### Synthesis, purification and identification of NAP

**Fig 2:**
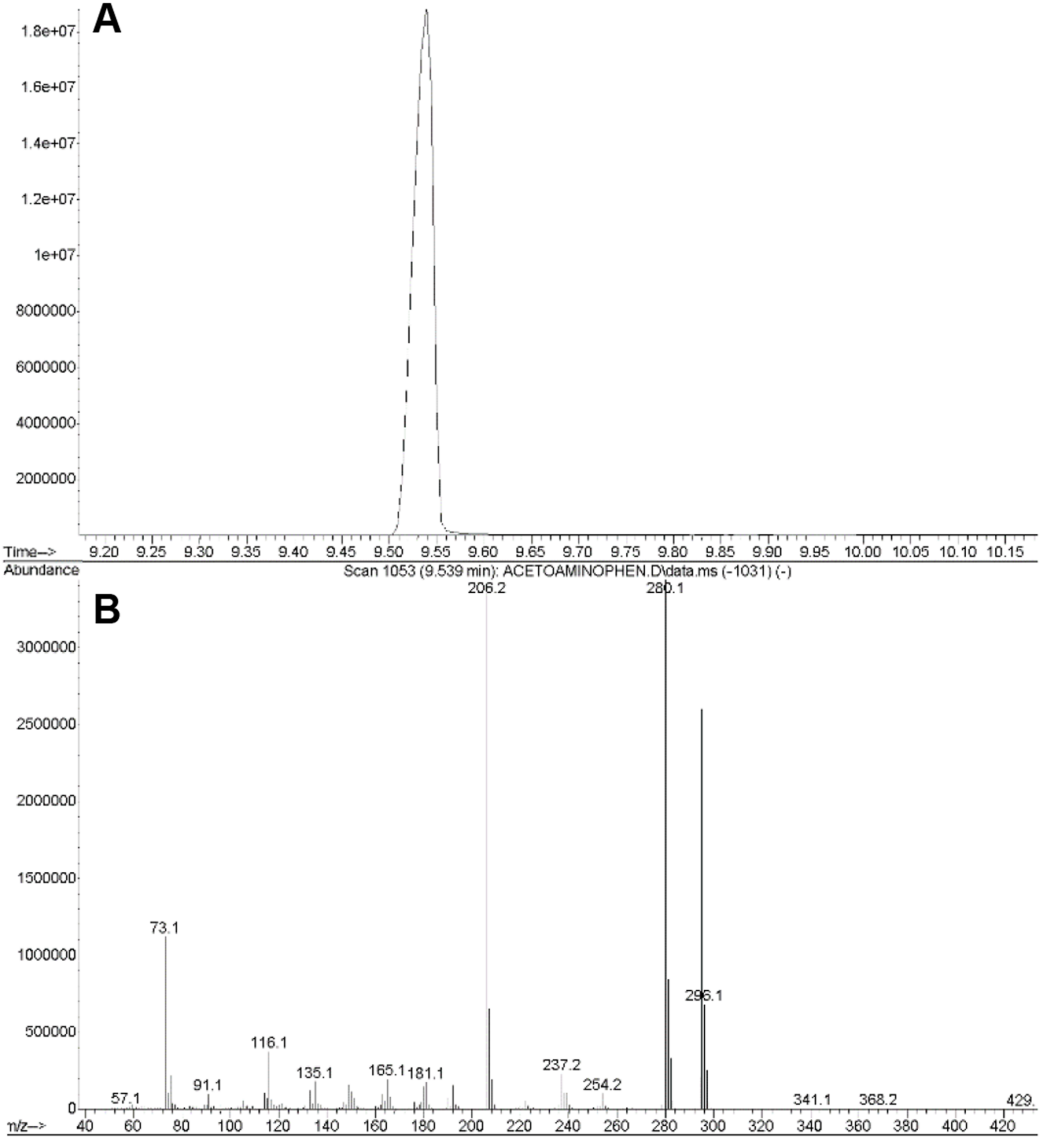
GC/MS spectra of paracetamol standard. Panel A: Gas chromatography trace. Panel B: Mass spectra trace. Data were acquired in the SCAN mode (50–800 amu). C: Matching of standard sample to National Institute of Standards and Technology (NIST) standard database. Panel A: Mass spectra and formula of paracetamol. Panel D: Mass spectra matching to NIST (top NIST, bottom standard). Data were acquired in the SCAN mode (50–800 amu).

**Fig 3:**
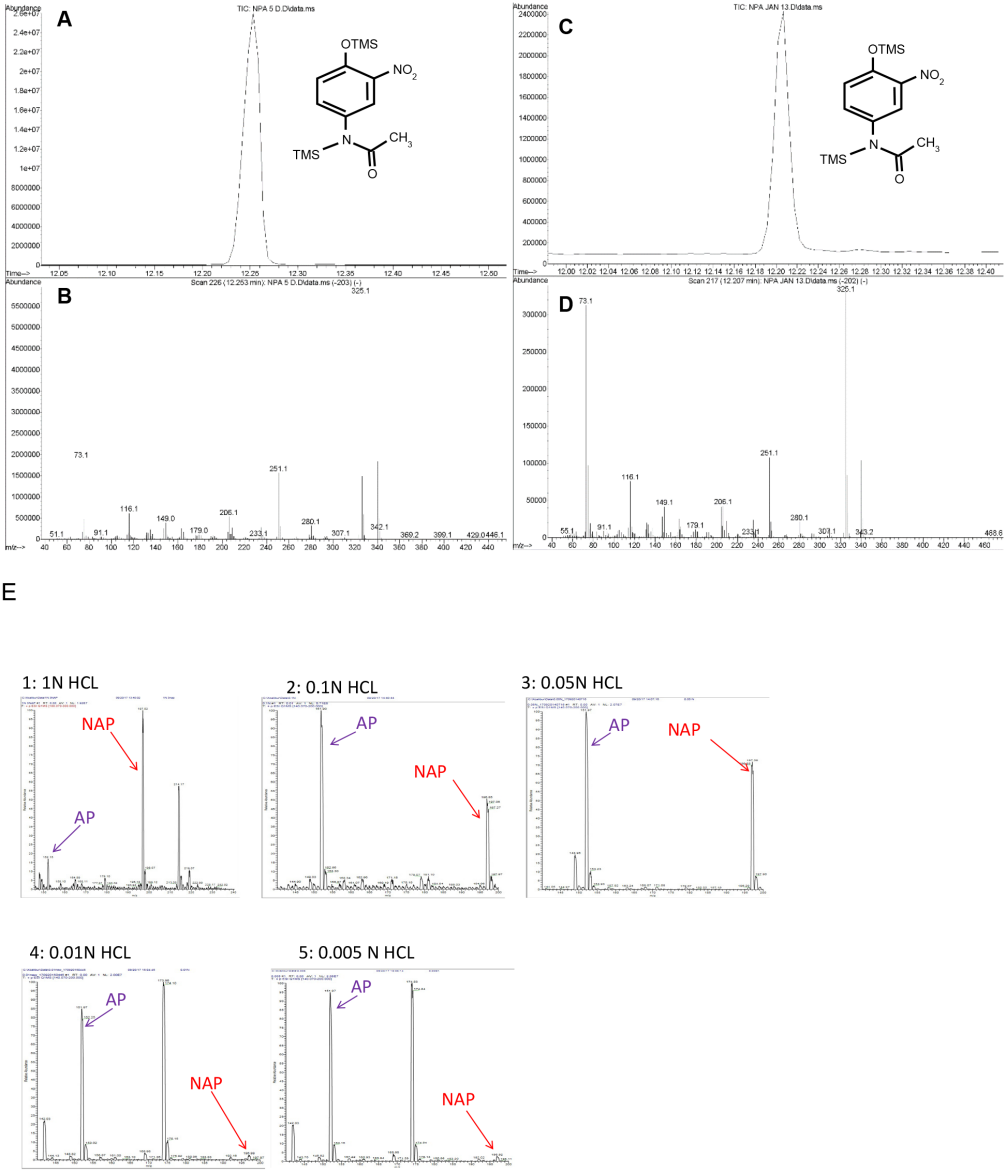
Determination and stability of 3-nitroacetaminophen (NAP)-TMS derivative product. Panels A and B: Gas chromatography and mass spectra traces, respectively, at time 0. Panels C and D: Gas chromatography and mass spectra traces, respectively, at 3-month time point. Data were acquired in the SCAN mode (50–800 amu). TMS(trimethylsilyl). Panel E demonstrates the LCMS trace peaks of NAP (arrow) post when the AP nitration was done in the presence of 1N, 0.1N, 0.5N, 0.01N, 0.05N and 0.005N HCL.

NAP was produced by nitration of AP in nitrite at pH 4, 2, and 0.3 and purified by HPLC with 15 minutes of elution time in a mobile phase of 20 mM ammonium acetate and 5% acetonitrile. The NAP peak eluted at 8 minutes (n = 10 replicates, Fig 1). NAP was identified by GC/MS (Fig 2A and 2B, representing 3 replicates). The MS profile was similar to one previously published by Lakshmi, et al^32^. As a standard analytical quality AP was converted to its trimethylsilyl (TMS) derivative and analyzed by GC/MS. Fig 2C and 2D shows GC/MS trace with prominent molecular ion 296 and typical loss of CH_3_^+^ ion 280. Fig 3 shows validation of AP TMS derivative by NIST database matching (98%; n = 3 replicates). Fig 3 shows the NAP TMS derivative. Panels A and B show GC/MS spectra matching data published by Lakshmi et al [32]. Molecular ion 340 corresponds to NAP TMS molecular weight and ion 325 representing loss of CH_3_^+^ group. Panels C and D show the same samples analyzed after 3 months of storage of lyophilized NAP. The relative signal was 10% of the original but usable for identification and quantification. To determine the effect of HCL concentration on NAP synthesis AP reactions were done using HCL covering. The AP nitration reactions were done using HCL concentrations of 1N, 0.1N, 0.05N, 0.01N or0.005N (panel E1-5). AP nitration and generation of NAP was evident at 0.05N HCL and above demonstrating the concentration dependent impact of HCL concentration on the synthesis of NAP.

### Conjugation of Der p1 with NAP

Haptens are electrophilic and can directly react with protein nucleophiles [37]. Amino acids with the most-reactive nucleophilic side chains are lysine, cysteine and histidine [38]. We expected covalent binding of NAP to Der p1 because of the reactivity of the electrophilic moiety on NAP with the nucleophilic amino acids on Der p1. Conjugation was accomplished by incubating 25μg of Der p1 with 50μg of NAP at 37°C for one hour at pH 7.4, 6.0 and 4.0. No significant difference in reaction results resulted from changes in pH. LC-MS analysis showed a molecular mass shift of 194.03Da in the NAP + Der p1 groups as compared to untreated Der p1 of (Table 1) (n = 3; p<0.05). The modification was mapped to Der p1 Cysteine (Cys) 129 or 132 (there were insufficient ion fragments to distinguish between these sites), however, the Cys 129 in native Der p1 forms a disulfide bond with Cys 169 and is unlikely the binding site absent denaturation [39] making Cys132 more likely.

**Table 1:** Liquid chromatography-mass spectrometry analysis of Der p1/3-nitro-acetaminophen (NAP) conjugate. LC-MS analysis of NAP + Der p1 conjugation reaction at pH7 showing mass shift in the NAP treated group. CAM (carbamidomethyl Cys) OxMet (oxidized methionine), Mw (Molecular weight), Obs (observed), Cal (calculated).

Der p1 Peptide	No	Site		Mw(Obs.)	Mw(Cal.)	Mass error	
						(ppm)	
MQGGCGSCWAFSGVAATESAYLAYR	125–149	C129 or	Un-modified	2643.1622	2643.1425	7	CAM
		C132	Der p1 peptides				
				2659.131	2659.1374	-2	CAM
							OxMet
				2715.163	2715.1516	4	2CAM
							OxMet
			NAP-modified Der p1	2780.1564	2780.1538	1	NAP
			peptides				
				2796.1545	2796.1487	2	NAP
							OxMet
				2837.1546	2837.1752	-7	NAP
							CAM

### Immunoreactivity of the NAP and Der p1 compounds

**Fig 4:**
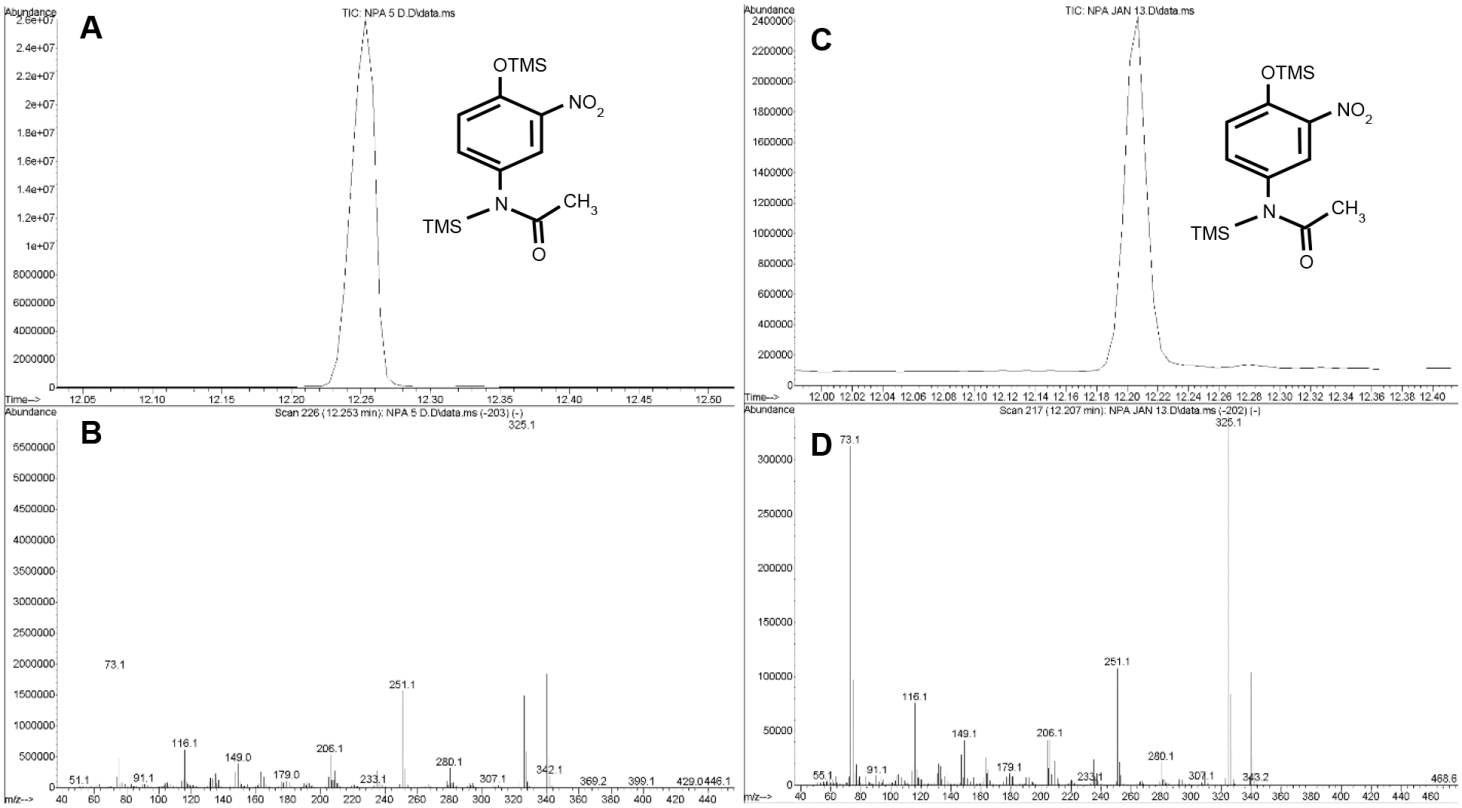
Peripheral blood monocyte (PBMC) response to Der p1 with and without 3-nitro-acetaminophen (NAP). PBMCs were obtained from 7 different individuals and cultured without treatment (Blank), after stimulation with acetaminophen (AP; 100ng/ml), 3-nitroacetaminophen (NAP; 50ng/ml), Der p1 (Drp; 50ng/ml), Der p1 conjugated with NAP (N+D; 50ng/ml), Der p1 mixed with NAP (N/D; 50ng/ml), Der p1 conjugated with AP (A+D; 50ng/ml), and Der p1 mixed with AP (A/D; 50ng/ml). After 24 hours PBMC cytokine (pg/mL) and adenosine triphosphate (ATP, mol/mL) levels were quantified as a measure of cell proliferation. ATP levels were standardized to subject baseline to account for intrasubject variability. Der p1 conjugated to NAP induced a significant response in IL-6 (Fig 4A), IL-1β (Fig 4B) and ATP (Fig 4D). There was no significant difference in any other cytokine with TNFa shown as reference (Fig 4C).

To determine if NAP conjugated Der p1 enhances immune response to Der p1, we tested Der p1 with and without conjugation with NAP using human PBMCs in vitro monitoring cytokine response and cellular activity (Fig 4). The PBMC response to NAP versus NAP conjugated to Der p1 demonstrated very different responses. NAP conjugated to Der p1 induced a significant IL-6 and IL-1β response (pg/mL, n = 7; p<0.05) (Fig 4A, 4B & 4C), which did not occur when unconjugated NAP was added concurrently with the Der p1. Further, the conjugation of Der p1 with NAP did not appear to have a significant impact on the other cytokines measures. PBMC’s were responsive to the nonspecific stimulant PHA (positive control). NAP conjugated Der p1 induced a proliferative response in the monocytes compared to the unstimulated sample (Fig 4D n = 3; p<0.05) with a focus on PBMC ATP production. The NAP combined with the Der p1 had the same impact on PBMC proliferation and ATP production as NAP conjugated to the Der p1. No other sample generated a significant response.

## Discussion

The original hypothesis that increased prevalence of asthma was related to AP use was proposed in 1998 [40]. Reports subsequently suggested an association between AP use and rhinoconjunctivitis, eczema and asthma severity [2]. More recently, the NHLBI AsthmaNet AVICA Trial found no difference in asthma incidence in children treated with either AP or ibuprofen for fever from the age of 3–5 [8]. However, the original ISAAC study involved AP exposure in children less than 1, and Sordillio et al, among others, showed prenatal AP exposure was associated with wheeze. The effects of early exposure, and the association of AP use with atopic disease, remain unclear.

Nitration is the addition of NO_2_^+^ to a target. Multiple nitration pathways exist in inflamed airways. The reaction of nitric oxide (NO) with superoxide (O_2_^-^) forms peroxynitrite (OONO^-^) which, when protonated forms peroxynitrous acid a potent nitrating agent [41,42]. Additionally, abundant airway nitrite (NO_2_^-^), when protonated forms nitrous acid, another nitrating agent [43]. Eosinophil peroxidase and neutrophil myeloperoxidase enzymatically nitrate proteins [44]. 3-nitro-tyrosine is detected in inflamed aiways [20,45], exhaled breath condensate analysis [46–48] induced sputum, and airway lavage samples [21,22,24,49]. AP is uniformly distributed in most tissues [50] and detected in rat lungs [51]. Under acidic conditions found in the stomach and inflamed airways, we demonstrate AP, like tyrosine, can be nitrated to form NAP.

Up to 85% of asthmatics are sensitized to house dust mites [52,53]. *Dermatophagoides pteronyssinus* is a predominant HDM species [54] with Der p1 being its principal allergen. We demonstrate AP covalently binds to Der p1 at Cys 132. When PBMC are exposed to NAP conjugated Der p1, the PBMC are stimulated to proliferate and produce IL-1, and IL-6. Interestingly, the cytokine response was not type 2 nor was there a strong response to Derp1 alone. It has been shown that mutation of Cys 132 leads to loss of the enzymatic activity [41] implicated in the type 2 responses to Der p1 [55]. The NAP binds to this same enzymatic active site of Der p1, potentially similarly affecting immune bias.

Immunologic response to haptenized proteins may be directed at the native protein, the protein-hapten complex, or the hapten. The cytokine response was only apparent in the context of conjugation between the NAP and Der p1. The PBMC proliferative response, as monitored by the production of ATP, was similar whether the NAP was conjugated to the Der p1 or not. The combined presence of both NAP and Der p1 enhanced PBMC ATP production compared to when the PBMCs treated with NAP or Der p1 independent of each other. The difference between the cytokine levels and the ATP response suggests that the ATP response to NAP and Der p1 is exquisitely sensitive and may reflect the overall sensitivity of the PBMCs to both NAP and Der p1 in which ATP is a product of immediate activation of the cells. The subsequent cytokine production involves a variety of down-stream events to cellular activation which are reflected in the specificity of the response to the NAP conjugated to Der p1. We speculate that immune responses to a given protein may differ based on whether the protein of interest is nitrated. A patient’s allergic immune response could be influenced by the nitration status at time of initial presentation.

In summary, AP is nitrated under acidic conditions and reacts with Cys 132 on Derp1 forming a hapten-antigen complex capable of amplifying human monocytic proliferative responses to antigen stimulation. Acidic nitrating conditions—perhaps particularly in the presence of AP—may modify antigenicity and human immune responses.
